# Neutrophil-derived heparin binding protein triggers vascular leakage and synergizes with myeloperoxidase at the early stage of severe burns (With video)

**DOI:** 10.1093/burnst/tkab030

**Published:** 2021-09-17

**Authors:** Lu Liu, Yiming Shao, Yixuan Zhang, Yunxi Yang, Jiamin Huang, Linbin Li, Ran Sun, Yuying Zhou, Yicheng Su, Bingwei Sun

**Affiliations:** School of Medicine, Jiangsu University, Zhenjiang 212001, Jiangsu Province, China; Department of Burns and Plastic Surgery, Affiliated Suzhou Hospital of Nanjing Medical University, Suzhou 215002, Jiangsu Province, China; Department of Burns and Plastic Surgery, Affiliated Suzhou Hospital of Nanjing Medical University, Suzhou 215002, Jiangsu Province, China; School of Medicine, Jiangsu University, Zhenjiang 212001, Jiangsu Province, China; School of Medicine, Jiangsu University, Zhenjiang 212001, Jiangsu Province, China; Department of Burns and Plastic Surgery, Affiliated Suzhou Hospital of Nanjing Medical University, Suzhou 215002, Jiangsu Province, China; Department of Burns and Plastic Surgery, Affiliated Suzhou Hospital of Nanjing Medical University, Suzhou 215002, Jiangsu Province, China; Department of Burns and Plastic Surgery, Affiliated Suzhou Hospital of Nanjing Medical University, Suzhou 215002, Jiangsu Province, China; Department of Burns and Plastic Surgery, Affiliated Suzhou Hospital of Nanjing Medical University, Suzhou 215002, Jiangsu Province, China; Department of Burns and Plastic Surgery, Affiliated Suzhou Hospital of Nanjing Medical University, Suzhou 215002, Jiangsu Province, China; Department of Burns and Plastic Surgery, Affiliated Suzhou Hospital of Nanjing Medical University, Suzhou 215002, Jiangsu Province, China

**Keywords:** Severe burn, Heparin binding protein, Myeloperoxidase, Vascular leakage, Glycocalyx

## Abstract

**Background:**

Burn shock caused by vascular leakage is one of the main causes of high mortality in severe burn injury. However, the pathophysiological mechanism of vascular leakage is still unclear. The purpose of this study was to explore the molecular mechanism of vascular leakage in the early stage of severe burn and provide a new target for the treatment of severe burns.

**Methods:**

Neutrophils were isolated from human peripheral blood by magnetic beads sorting. ELISA was used to detect neutrophil-derived granule proteins and glycocalyx injury products in plasma. The vascular leakage and neutrophil movement were assessed by *in vivo* laser confocal imaging in mice, and high-quality video were provided. Adhesion-related molecules were investigated by qRT-PCR. The damage to glycocalyx of mice vascular endothelial cells was observed by transmission electron microscope and scanning electron microscope. Proteomic analysis, flow cytometry and immunofluorescence were used to further study the relationship between human peripheral blood neutrophil-derived hypochlorite (HOCl) and CD44 of human vascular endothelial cells.

**Results:**

In this study, we found that rapidly increasing activated neutrophils secrete heparin binding protein (HBP) and myeloperoxidase (MPO) after severe burn injury. Increased HBP triggers vascular leakage with synergy of MPO, results in systemic edema and burn shock. Furthermore, we found that the MPO catalytic product HOCl but not MPO triggers CD44 extracellular domain shedding from vascular endothelial cells to damage the glycocalyx. Damage to the glycocalyx results in firm adhesion of neutrophils and increases vascular leakage. However, MPO inhibitors partially protect the glycocalyx of vascular endothelial cells. The combination of HBP and MPO inhibitors markedly reduces vascular leakage and systemic edema in the early stage of severe burns.

**Conclusions:**

Taken together, these data reveal that neutrophil-derived HBP and MPO play an important synergies role in triggering vascular leakage at the early stage of severe burns. Targeted intervention in these two biomolecules may introduce new strategies for helping to reduce large amount of fluid loss and subsequent burn shock.

Highlights

- Rapidly increasing activated neutrophils secrete HBP and MPO after severe burn injury.
- MPO catalytic product HOCl triggers CD44 extracellular domain shedding from vascular endothelial cells to damage the glycocalyx.
- Damaged glycocalyx results in firm adhesion of neutrophils and increases vascular leakage.
- Increased HBP triggers vascular leakage with synergy of MPO, results in systemic edema and burn shock.

## Background

Burns are one of the most common wounds worldwide, and tens of millions of people seek treatment for burns every year [1]. Burn mortality is associated with burn size, burn depth, age and smoke inhalation. Data from the United States show that when the burn size exceeds 70% of the total body surface area (TBSA), the mortality rate is 69% [2]. Shock occurs rapidly after burn injury, which is mainly due to the increase in vascular leakage and low blood volume caused by the transfer of fluid from the blood vessels to the interstitial space [3]. If fluid resuscitation is not performed in time, insufficient blood volume will lead to a decrease in cardiac output and cardiogenic shock. Moreover, almost all severe burn patients will have systemic edema within 24–48 h after injury, which will increase systemic vascular resistance, hinder blood perfusion, and put shock patients into a vicious cycle [4].

The surface of the vascular endothelium is covered with a glycocalyx layer composed of hyaluronic acid (HA), glycoprotein, sulfate proteoglycan and some plasma proteins. Previous studies have suggested that glycocalyx is mainly used as a capillary exchange regulator, sensing fluid shear stress and regulating the inflammatory response [5–7]. A later study showed that endothelial glycocalyx loss can cause neutrophil adhesion to increase, and these attached neutrophils can further cause neutrophil inflammation. The authors found that this glycocalyx loss is an active regulation of vascular endothelial cells through endogenous heparanase after sensing circulating pathogen-associated molecular patterns. The loss of the surface layer of endothelial cells exposed the adhesion molecules including intercellular adhesion molecule-1 (ICAM-1) and vascular cell adhesion molecule-1 (VCAM-1), allowing neutrophils to adhere to the endothelial surface for molecular recognition. This provides a new perspective on other research to understand the interaction between glycocalyx and neutrophils [8]. Other studies have shown that glycocalyx dysfunction may be associated with vascular leakage due to burns [9–13]. This suggests that there may be different interactions between neutrophils and glycocalyx in different disease settings. In severe burns, further research on this issue is more important.

Although the emergence of various new dressings and local immunotherapy in the past two decades has significantly promoted wound treatment in severe burn patients, there has been no breakthrough in the treatment of vascular leakage or systemic edema during the early stage of severe burns [14–16]. In this study, we verified the induction of vascular leakage by neutrophil-derived heparin binding protein (HBP) in the early stages of severe burns. Additionally, we investigated a new mechanism of neutrophil-derived myeloperoxidase (MPO) damage to glycocalyx of vascular endothelial cells, which is different from previous findings in sepsis [11, 17]. We found that the MPO catalytic product HOCl but not MPO triggers CD44 extracellular domain shedding from vascular endothelial cells to damage the glycocalyx. The transmembrane CD44 binds to HA [7], and the shedding of CD44 is related to the loss of HA [18]. Our findings suggest that neutrophil-derived HBP and MPO could induce vascular leakage in different ways in the early stage of severe burns and eventually lead to systemic edema. This study provides new targets for the early treatment of severe burns.

## Methods

### Experimental design

*In vitro* experimental design: The concentration of HBP, MPO, neutrophil elastase (NE), matrix metallopeptidase-9 (MMP9), HA, heparin sulfate (HS) and syndecan-1 (SDC-1) in plasma of severe burn patients, healthy volunteers and rats were detected by ELISA. The peripheral blood neutrophils chemotaxis of severe burn patients and healthy volunteers were detected by agarose chemotaxis model. Flow cytometry was used to detect the reactive oxygen species (ROS) and apoptosis of peripheral blood neutrophils in patients with severe burn and healthy volunteers. CD44 in human microvascular endothelial cells was also detected by flow cytometry. Monolayer human microvascular endothelial cells (HMEC-1) were cultured in 24-well Transwell systems for permeability test. Caspase-3, caspase-8, caspase-9, ICAM-1 and VCAM-1 in HMEC-1 cells were detected by reverse transcription PCR. Plasma samples from 6 severely burned patients and 3 healthy volunteers were analyzed by proteomic analysis. Scanning electron microscope was used to detect the surface of abdominal wall vein and femoral vein in mice. The glycocalyx of femoral vein in mice was detected by transmission electron microscope. The CD35 and CD63 molecules, cytoplasmic ROS and HOCL of neutrophils in the peripheral blood of healthy volunteers after immunofluorescence staining were detected by a laser confocal microscope. The plasma total protein and albumin of burn-injured rats and sham group were detected by automatic biochemical analyzer. The number and proportion of neutrophils in peripheral blood of severely burned patients, healthy volunteers, burn-injured mice and sham operated mice were detected by hematology analyzer.

*In vivo* experimental design: The blood vessels of burn-injured mice and sham operated mice pre-injected with fluorescein dextran were observed by laser confocal microscopy. The wet to dry ratio of lung, spleen, kidney, liver and heart of mice pre-injected with Evans blue was detected. Laser confocal microscopy was also used to observe the expression of CD44 in the vascular endothelial cells of the abdominal wall of mice treated with HOCl.

### Data collection

A total of 15 severe burn patients were enrolled in this study. For each patient, the following data were collected: age, sex, degree of burn and TBSA, total fluid input, urine output, mean venous pressure (MAP), shock index, as well as neutrophils count (Table 1).

**Table 1:** Demographic and clinical characteristics of severe burn-injured patients and healthy volunteers

	**Patient demographics**	
**Characteristic**	**Healthy controls (n = 15)**	**Burn-injured patients (n = 15)**
Age, y (IQR)	38 (26, 53)	45 (33,55)
Gender, (M: F)	10: 5	12: 3
%TBSA	—	51 (26,76)
Degree of burn	—	II ~ III
Inhalation injury (Y: N)	—	8: 7
Complication (Y: N)	—	6: 9
Total fluid input (1st 48 h) (mL)	—	7077 (5145, 9307)
Urine output (1st 48 h) (mL)	—	2756 (2020, 2830)
MAP (mmHg)	92.3 (75.4, 99.6)	95.3 (86.1, 100.7)
Shock index	—	0.69 (0.59, 0.78)
Neutrophils count (×10^9^/L)	7.4 (6.93, 8.17)	17.94 (12.32, 22.16)
Survived (Y: N)	—	15: 0

Data are presented as median (IQR). Complications from this table include acute kidney injury, pulmonary blast injury, and hypoxic ischemic encephalopathy *y* year, *IQR* interquartile range, *M* male, *F* female, *TBSA* total body surface area, *Y* Yes, *N* No

### Animals

Pathogen-free male C57BL/6 mice aged 6–8 weeks and weighing 18–20 g and pathogen-free male SD rats aged 6–7 weeks and weighing 230–250 g were purchased from Zhaoyan New Drug Research Center (Suzhou, China). Before beginning the experiment, the animals were maintained in a specific pathogen-free environment with free access to water and food for 1 week to adapt to the experimental conditions. This study was approved by the Medical Ethical Committee of Nanjing Medical University. For experiments involving human blood samples, signed informed consent was obtained from all healthy volunteers. Blood samples were taken from the cubital veins of healthy donors. All the experimental methods were carried out in accordance with the approved guidelines. All experimental procedures involving mice were carried out in strict accordance with the recommendations in the Guide for the Care and Use of Laboratory Animals of the National Institutes of Health and State Key Laboratory of Pathogens and Biosecurity of the Institute of Microbiology and Epidemiology.

### Burn model

Before model induction, 8% chloral hydrate was injected intraperitoneally for anesthesia. After 20 min, the mice or rats were observed and anesthetized. After the mice or rats were fixed properly, 20% TBSA for mice or 30% TBSA for rats was measured with tinfoil, and a full-thickness burn was induced with boiling water for 10 seconds (rats 13 seconds) [19]; the sham operation group was treated with water at 37°C. Ringer’s solution was used for rehydration, and the total rehydration was calculated according to Parkland formula (1.5 mL × TBSA × kg), one half was injected intraperitoneally immediately after injury, and one fourth was injected intraperitoneally at 4 h and 8 h after injury, respectively.

### ELISA

Blood samples of humans or rats containing the anticoagulant EDTA were centrifuged at 3000 g for 10 min at room temperature, and then the upper two-thirds of the plasma were carefully removed for testing. The samples were centrifuged at 500 g for 5 min, and the upper two-thirds of the cell culture supernatant were carefully aspirated for testing. The experimental steps were performed according to the instructions of the corresponding ELISA kit. The following kits were used: human HBP ELISA kit (Thermo Fisher, USA), human MPO ELISA Kit (R&D Systems, USA), human elastase ELISA kit (R&D Systems, USA), human MMP9 ELISA kit (R&D Systems, USA), human hyaluronic acid ELISA kit (Biorbyt, UK), human heparan sulfate ELISA kit (Biorbyt, UK), human SDC-1 ELISA kit (Biorbyt, UK), rat HA ELISA kit (Cusabio, China), rat HS ELISA kit (Cusabio, China), and rat SDC-1 ELISA kit (Cusabio, China).

### Flow cytometry

Human peripheral blood neutrophils were isolated using a neutrophil isolation kit (Miltenyi Biotec, Germany). To measure apoptosis, antibodies, Annexin V and 7-AAD (BD Bioscience, USA) were added according to the manufacturer’s protocol. After 15 min of incubation in the dark at room temperature, neutrophils were evaluated. HMEC-1 cells were cultured with endothelial cell culture medium (ECM) (ScienCell, USA) until complete fusion. To measure the expression of CD44 in HMEC-1 cells, PE-Cy7-conjugated CD44 antibodies (Abcam, UK) were added according to the manufacturer’s protocol. After 15 min of incubation on ice in the dark, neutrophils were evaluated. AZD5904 (MCE, USA), sivelestat (MCE, USA), ilomastat (MCE, USA), recombinant human MPO (Novoprotein, China), recombinant human HBP (Novoprotein, China), recombinant human MMP9 (Abcam, UK), recombinant human HBP (Novoprotein, China), and native human NE (Abcam, UK) were used.

### Reverse transcription PCR

Total RNA was extracted with TRIzol reagent (Invitrogen, USA). Complementary DNA was synthesized from 1 μg of total RNA by reverse transcription, and the mRNAs of interest were quantified by qRT-PCR using SYBR premix (Takara, Japan) on a BioRad CFX96. GAPDH was selected as the reference gene (Table 2).

**Table 2:** Primers used to amplify mRNAs via qRT-PCR

**Gene**	**Forward primer (5′-3′)**	**Reverse primer (5′-3′)**
Caspase-3	CATGGAAGCGAATCAATGGACT	CTGTACCAGACCGAGATGTCA
Caspase-8	CGAAGGTGCTACCATCGTGA	GGTTCTTGCTTCCTTTGCGG
Caspase-9	CTCAGACCAGAGATTCGCAAAC	GCATTTCCCCTCAAACTCTCAA
CD44	CTGCCGCTTTGCAGGTGTA	CATTGTGGGCAAGGTGCTATT
SDC-1	CTGCCGCAAATTGTGGCTAC	TGAGCCGGAGAAGTTGTCAGA
ICAM-1	ATGCCCAGACATCTGTGTCC	GGGGTCTCTATGCCCAACAA
VCAM-1	GGGAAGATGGTCGTGATCCTT	TCTGGGGTGGTCTCGATTTTA
GADPH	GTGAAGGTCGGAGTCAACG	TGAGGTCAATGAAGGGGTC

### Immunofluorescence analysis

Human peripheral blood neutrophils were isolated using a neutrophil isolation kit (Miltenyi Biotec, Germany). To stain for CD35 (Abcam, UK) and CD63 (Abcam, UK), the cells were washed twice with ice-cold phosphate buffered saline (PBS), 4% paraformaldehyde was added, and the cells were fixed at room temperature for 15 min. The cells were washed twice again and blocked with 5% goat serum (Solarbio, China) for 1 h at room temperature. Then, the primary antibody (1:200) (Abcam, USA) and the secondary antibody (1:2000) (Abcam, USA) were sequentially added. Multiple washings were performed between each step, and in order to get a better washing effect, 0.05% Tween 20 was added into PBS. To stain for CD44_ECD_ and CD44_ICD_ [20], the cells were fixed with 4% paraformaldehyde and permeabilized with 0.01% Triton X-100 (Solarbio, China) for 30 min. HMEC-1 cells are cultured to near confluence in ECM. The cells were washed twice again and blocked with 5% goat serum (Solarbio, China) for 1 h at room temperature. Then, the CD44ECD/CD44ICD primary antibody (1:200) (Abcam, UK) and the secondary antibody (1:2000) (Abcam, UK) were sequentially added. After antibody incubation, PBS containing 0.05% Tween 20 was used for multiple washes. HMEC-1 cells were treated with recombinant human MPO (Novoprotein, China), recombinant human HBP (Novoprotein, China), hypochlorite (Aladdin, USA) and hydrogen peroxide (Aladdin, USA), and stained accordingly. To stain for ROS and HOCl, the cells were washed twice with ice-cold PBS, and the fluorescent probes for ROS (Sigma, USA) and HOCl (Sigma, USA) were directly added. After being incubated at 37°C for 30 min, the cells were washed twice again before being sealed and observed. Cells can survive for a short time without fixation.

### Endothelial cell permeability assay

Human microvascular endothelial cells (HMEC-1) (ATCC, USA) were cultured in endothelial cell culture medium (ScienCell, USA) supplemented with FBS, ECGS and P/S. HMEC-1 cells were seeded in 6.5 mm Transwell plates with 0.4 μm pore polycarbonate membrane inserts (Corning, USA). Fibronectin (Sigma-Aldrich, USA) was added during culture to achieve complete fusion. BSA-HRP (Solarbio, China) and recombinant HBP (Novoprotein, China) were added to the upper compartment of the Transwell chamber. After 30 min, a fixed volume of medium was collected from the lower chamber, and the OD value was measured at 450 nM by adding TMB substrate. The concentration of BSA was calculated with the BSA-HRP standard curve. Native cow aprotinin protein (Abcam, UK) was used.

### Neutrophil isolation

Human peripheral blood neutrophils were isolated using a neutrophil isolation kit (Miltenyi Biotec, Germany). The procedure was performed according to the protocol provided by the manufacturer. Previous studies have shown that neutrophil isolation by Ficoll density gradient centrifugation can trigger MPO release [21, 22], which should be avoided when studying neutrophil-related MPO.

### Thermal stimulated neutrophil model

Human peripheral blood neutrophils were isolated using a neutrophil isolation kit (Miltenyi Biotec, Germany). The cells were re-suspended in preheated RPMI-1640 medium (Gibco, USA). After reaching the set time, add 10 times the volume of room temperature medium for dilution to reduce the temperature to room temperature. After centrifugation at 400 g for 5 min, the supernatant was discarded and the cells were gently re-suspended in RPMI-1640 medium supplemented with 10% inactivated fetal bovine serum (Gibco, USA). According to the experimental requirements, the cells were continuously cultured in the incubator containing 5% carbon dioxide at 37°C.

### *In vivo* imaging of mouse blood vessels

The mice were anesthetized by intraperitoneal injection of 8% chloral hydrate, and the abdominal hair was removed after proper fixation. A 2-cm incision was made along the white line of the abdomen. After cutting the skin, blunt separation was performed on one side to free the skin with an area of approximately 2 × 1 cm. It is important to be very careful to avoid damaging the blood vessels during this operation. The separated skin was gently pulled at a 90-degree angle and observed under a Zeiss LSM 900 confocal microscope. Texas Red Dextran (Thermo Fisher, USA) was used as a plasma tracer, and mouse neutrophils were labeled with iFluor™ 647-streptavidin-conjugated (AAT Bioquest, USA) and biotin-conjugated anti-mouse Ly-6G (Biolegend, USA) or biotin-conjugated anti-mouse CD44 (Thermo Fisher, USA). Hyaluronidase (Sigma, USA) was used. All reagents were injected intravenously via the tail vein.

### Statistical analysis

All statistical analyses were performed and graphs were prepared with GraphPad Prism 8.0 software and Adobe Illustrator. The Shapiro-Wilk test was used to test the normality of continuous variables. The results are expressed as the mean ± standard deviation (SD). For group comparisons, one-way ANOVA was used for continuous variables with normal distribution. The Kruskal-Wallis test was used for continuous variables with skewed distributions. Tukey’s post hoc test or Dunn’s post hoc test was used for multiple comparisons. Student’s *t*-test and Wilcoxon paired signed-rank test were used to compare the differences between the two groups. Pearson correlation coefficient was used for correlation analysis. Statistical significance was set as *p* < 0.05.

## Results

### Systemic edema, neutrophil-derived HBP and MPO, and glycocalyx decomposition products were increased in the early stage of severe burns

**Figure 1.**
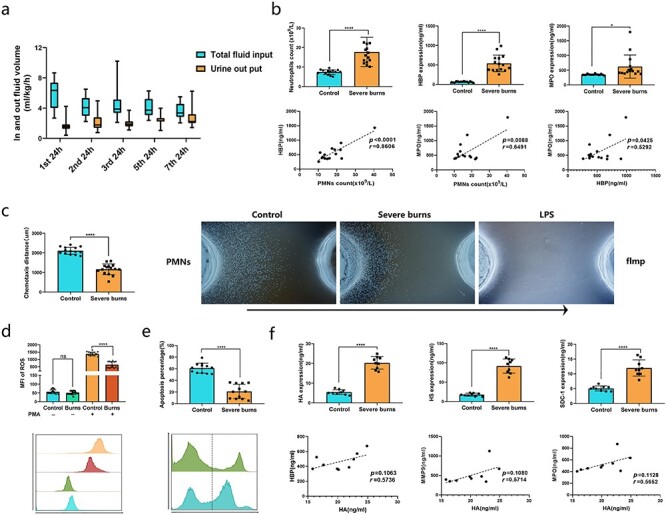
Systemic edema, abnormal neutrophil function and increased glycocalyx decomposition products in the early stage of severe burn patients. **(a)** The volume of fluid resuscitation and urine volume per hour after admission were closely monitored. The data on the first, second, third, fifth and seventh 24 h were selected for analysis. **(b)** Fifteen patients with TBSA of more than 30% and second- to third-degree burns were included in this study. The blood sample for testing was taken within 24 h of the injury. Each sample has undergone blood routine examination and ELISA test of HBP, MPO, NE and MMP9 to ensure that the data can be analyzed for correlation. n = 15 in each group. **(c)** Under agarose neutrophil chemotaxis model was used to detect the chemotaxis of neutrophils in different patients. Representative pictures from 15 independent experiments were selected for the present. n = 15. **(d)** Neutrophils were isolated from whole blood and incubated at 37°C for 30 min with or without 10 nM PMA. Then the ROS fluorescent probe was added and incubated at 37°C for 15 min. The continuous generation of ROS can be terminated by placing the sample into ice. The concentration of neutrophils was maintained at 5 × 10^6^/ml. n = 15. **(e)** Neutrophils were isolated from whole blood and cultured in a carbon dioxide incubator for 24 h. The percentage of annexin-V positive cells was detected by apoptosis detection kit. n = 12. **(f)** Each sample has undergone ELISA test of HA, HS and SDC-1 to ensure that the data can be analyzed for correlation. n = 9 in each group. All samples in Figure 1 are from healthy volunteers and burn patients. Data are mean ± SD; independent sample *t*-test was used to prove the difference between two groups. ^*^^*^^*^^*^p < 0.0001, ^*^^*^^*^p < 0.001, ^*^^*^p < 0.01, ^*^p < 0.05, compared with control group. *fmlp* formyl-methionyl-leucyl-phenylalanine, *HA* hyaluronic acid, *HBP* heparin binding protein, *HS* heparan sulfate, *LPS* lipopolysaccharide, *MMP9* matrix metalloprotein-9, *MPO* myeloperoxidase, *PMN* polymorphonuclear leukocyte, *SDC-1* syndecan-1

Our data show that fluid resuscitation within 48 h of the burn and the patient’s fluid input far exceeds urine output. These findings suggest that a large amount of fluid is transferred from blood vessels to tissue spaces in the early stage of severe burns, which is consistent with previous studies. Fluid input and urine output in the first 7 days were analyzed, and the most significant difference occurred during the first 24–48 h (Figure 1a). The demographic and clinical characteristics of the 15 severe burn patients are shown in Table 1.

The routine blood test results and ELISA analysis showed that the number and proportion of neutrophils and neutrophil-associated granule proteins, including HBP, MPO, NE and MMP9, were significantly increased in the early stage of severe burns (Figure 1b, and Figure S1a) [23, 24]. Correlation analysis showed that the number of neutrophils in plasma highly correlated with the expression of HBP and moderately correlated with MPO but not with NE or MMP9 in the early stage of severe burns. Correlation analysis of these granular proteins shows that only HBP and MPO had a moderate correlation (Figure 1b, and Figure S1b). The expression levels of plasma HBP and MPO (Figure S1c) in patients with minor injury (ISS ≤ 16) and severe injury (ISS > 16) [25] were analyzed by ELISA. The results showed that the expression levels of plasma HBP and MPO in patients with minor injury were not significantly increased, but severe injured patients had significantly increased levels. In addition to degranulation, other functions of neutrophils were abnormal in the early stage of severe burns. Neutrophil chemotaxis (Figure 1c) [26], ROS production (Figure 1d) [27, 28] and apoptosis were inhibited (Figure 1e) [29].

Other data showed that the plasma expression levels of HA, HS and SDC-1, which are the main decomposition products of glycocalyx [5, 12, 30], were also significantly increased in patients with severe burns in the early stage. No significant correlation was found between these glycocalyx decomposition products and HBP, MPO, NE or MMP9 (Figure 1f, and Figure S1d). There were some correlations between HBP and HA, MMP-9 and HA, and MPO and HA, but the correlations were not significant.

### HBP triggers vascular leakage and results in systemic edema

**Figure 2.**
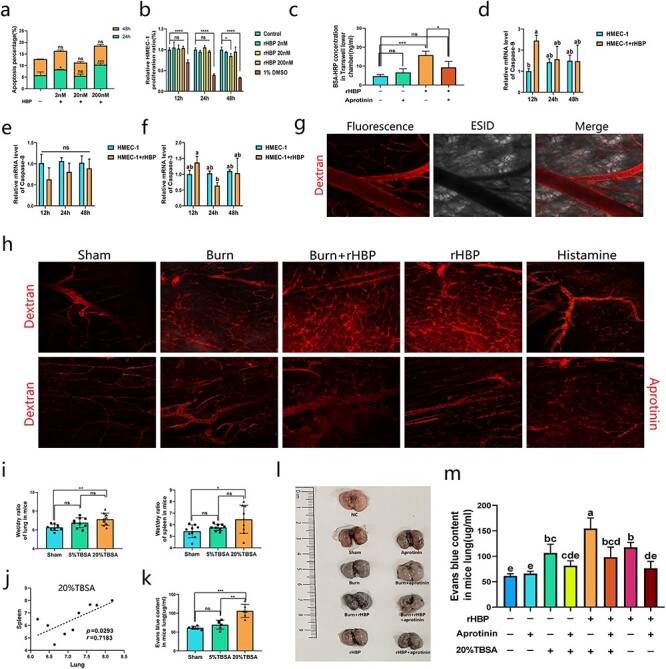
HBP triggers vascular leakage and results in systemic edema. **(a)** The 60% fused HMEC-1 cells were stimulated by rHBP at different concentrations of 2 nM, 20 nM (740 ng/ml) and 200 nM. The apoptosis of HMEC-1 cells was detected by flow cytometry at 24 h and 48 h, respectively. The data shows the proportion of 7AAD positive cells. The experiment was repeated three times. Each group was compared with the corresponding control. **(b)** HMEC-1 cells were cultured in 96-well plates with a density of 5 × 10^4^/well. The rHBP of 2 nM, 20 nM and 200 nM were added to intervene, and CCK-8 kit was used for detection. 1% DMSO group as positive control. The experiment was repeated three times. Each group was compared with the corresponding control. **(c)** After adding rHBP or aprotinin for 30 min, the BSA-HRP in the lower chamber of Transwell culture system was detected. BSA-HRP, 1 ug/ml; aprotinin, 20 ug/ml; rHBP, 20 nM. n = 6 in each group and compared with each other. **(d–f)** 20 nM rHBP was used to interfere with the unfused HMEC-1 cells for PCR analysis. n = 6 in each group. Each group was compared with control. **(g, h)** Texas Red dextran was injected through the tail vein of mice as a plasma tracer at a dose of 25 ug/g. Texas Red dextran, 70 000 MW; rHBP, 1.5 ug/g; histamine, 1 ug/g. All the observations lasted 60 min. Micrographs are representative of >9 separate experiments (Scale bar: 50 μm). **(i, j)** 24 h after the establishment of the model, the lung, liver, kidney, spleen and heart of the mice were obtained and the wet weight was recorded. Dry weight was obtained after drying at 150°C for 12 h. The left lungs were used for this analysis, the right lungs were used for Evans blue quantitative analysis. n = 9 in each group and compared with each other. **(k)** Evans blue in the right lung of mice in each group was extracted and compared according to the published protocol. n = 9 in each group and compared with each other. **(l, m)** Evans blue solution (50 μl of a 30 mg/ml solution in 0.9% normal, unheparinized saline) was injected via tail veins of mice. Aprotinin, 2.5 ug/g. n = 6 in each group. Photo is representative of 6 separate experiments. ANOVA with Tukey’s test. ^*^^*^^*^^*^p < 0.0001, ^*^^*^^*^p < 0.001, ^*^^*^p < 0.01, ^*^p < 0.05; a, b, c, d: different letters represent significant differences between the two groups, *p* < 0.05. *HBP* heparin binding protein, *HMEC-1* human microvascular endothelial cells, *rHBP* recombinant HBP, *TBSA* total body surface area, *BSA-HRP* HRP labeled bovine serum albumin

A previous study demonstrated that neutrophil-derived HBP increases endothelial permeability *in vivo* and *in vitro* [31]. We tested the effects of different concentrations of recombinant HBP (rHBP) on HMEC-1 cells (Figure 2a, b). The data showed that 20 nM rHBP had no significant effect on HMEC-1 cell apoptosis or proliferation at 24 h. At this dose, rHBP could significantly increase the permeability of human microvascular endothelial cell monolayer; however, aprotinin could partially antagonize this effect (Figure 2c, and Figure S2a) [32]. PCR analysis showed that 20 nM HBP could significantly upregulate the transcription of caspase-9 in HMEC-1 cells at 12 h, and there were no significant differences at 24 and 48 h (Figure 2d). The same dose of rHBP had no significant effect on caspase-8 or caspase-3 transcription (Figure 2e, f). Microscopic imaging of blood vessels in mice showed obvious vascular leakage in burn-injured or rHBP-injected mice, while worsened vascular leakage was observed in rHBP-injected mice with burn injury. Aprotinin significantly antagonized vascular leakage (Figure 2g,h, and Figure S2b) (Videos S1, S2, S3, and S4). Histamine was used as a positive control [33]. HE staining of some important organs of the burn-injured mice showed that compared with the sham group, the lungs, spleen, liver and kidneys of the burned mice had obvious edema, except for the heart (Figure S2d).The wet/dry ratio of the parenchymatous organs in burn-injured mice was determined (some data are not shown in the figure). The data showed that compared with those of the sham group, only the wet/dry ratios of the lung and spleen were significantly different, and the difference in the wet/dry ratio of the lung was more obvious than that of the spleen (Figure 2i). In the 20% TBSA group, there was a moderate correlation between the lung and spleen wet/dry ratios, while in the 5% TBSA or sham group, there was no correlation (Figure 2j, and Figure S2c). The results of the Evans blue staining assay showed that there was significant vascular leakage and plasma albumin transfer in the lung during the early stage of severe burns (Figure 2k) [34, 35]. Compared with the sham group, the rHBP group had significant vascular leakage, and the burn-injured rHBP-injected group had more severe vascular leakage than the burn-injured group. Compared with that of the burn-injured rHBP-injected group, aprotinin significantly reduced vascular leakage. Compared with that of the burn-injured group, aprotinin also reduced vascular leakage, but the effect was not significant (Figure 2l, m).

### Neutrophils secrete HBP and MPO after transient thermal stimulation. Neutrophil-derived HBP triggers vascular leakage, while MPO increases it

**Figure 3.**
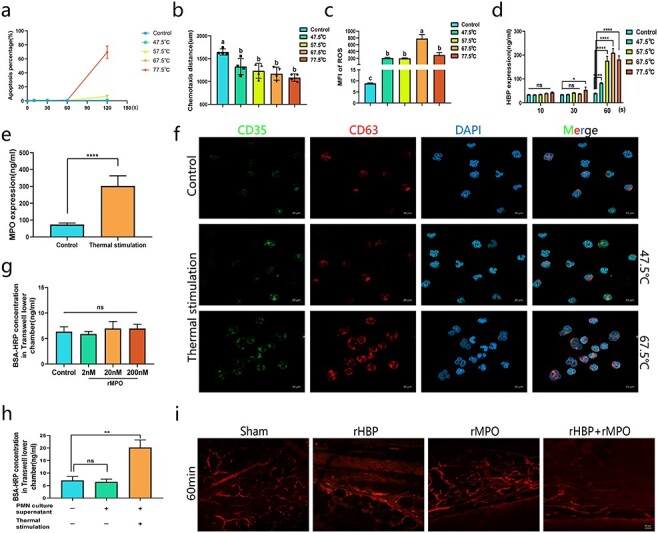
Neutrophils secrete HBP and MPO to trigger vascular leakage after transient heat stimulation. **(a)** After heat stimulation at different temperatures, human peripheral blood neutrophils were continuously cultured for 4 h. Cell concentration: 1 × 10^6^/ml. The data shows the proportion of 7AAD positive cells. **(b–e)** Thermal stimulation condition: 67.5°C for 60 seconds. Continue in incubator for 1 h, and use the cells or supernatant for corresponding detection. Cell concentration: 1 × 10^6^/ml. n = 4. Data are mean ± SD; n = 4. ANOVA with Tukey’s test. ^*^^*^^*^^*^p < 0.0001, ^*^^*^^*^p < 0.001, ^*^^*^p < 0.01, ^*^p < 0.05; a, b: different letters represent significant differences between the two groups, *p* < 0.05. **(f)** Thermal stimulation lasts for 60 seconds in the 47.5°C and 67.5°C groups. Micrographs are representative of >6 separate experiments (Scale bar: 10 μm). **(g, h)** After adding different treatment cell supernatants or different concentrations of rMPO for 30 min, the content of BSA-HRP in the lower chamber of the Transwell culture system was detected**.** PMNs culture supernatant: neutrophils were re-suspended by using 37.5°C medium for 60 seconds, and continued to culture for 1 h. Thermal stimulation: neutrophils were re-suspended by using 67.5°C medium for 60 seconds, and continued to culture for 1 h. BSA-HRP, 1 ug/ml. Data are mean ± SD; n = 6 in each group and compared with control. ANOVA with Tukey’s test. ^*^^*^p < 0.01. **(i)** rHBP, 1.5 ug/g mice weight; rMPO, 1 ug/g mice weight. rHBP and rMPO were diluted with normal saline and injected through tail vein. 1 mL Ringer’s solution was injected intraperitoneally. Micrographs are representative of >6 separate experiments (Scale bar: 50 μm). *BSA-HRP* HRP labeled bovine serum albumin, *HBP* heparin binding protein, *MPO* myeloperoxidase, *PMN* polymorphonuclear leukocyte, *rMPO* recombinant MPO

To study the effect of burn injury on neutrophils, we established a stable transient thermal stimulation model. Neutrophil apoptosis was analyzed for 4 h and showed that a single stimulation for less than 60 seconds did not induce significant neutrophil apoptosis (Figure 3a). When the stimulation time was fixed at 60 seconds, with increasing stimulation temperatures, the chemotactic distance was significantly shortened (Figure 3b), and the production of ROS was increased (Figure 3c). The expression of HBP increased continuously with increasing temperature and stimulation time and reached a maximum at 67.5°C and 60 seconds (Figure 3d). The expression of MPO in the culture supernatant was also significantly increased after heat stimulation at 67.5°C for 60 seconds (Figure 3e). CD35 and CD63 are markers of secretory vesicles and azure granules, respectively. Immunofluorescence analysis showed that the expression of CD35 and CD63 was upregulated in the neutrophil membrane after heat stimulation (Figure 3f). Different concentrations of recombinant MPO did not increase the permeability of vascular endothelial cells (Figure 3g). Neutrophils were stimulated by heat, then cultured in an incubator for 1 h. Neutrophil supernatant significantly induced the permeability of vascular endothelial cells (Figure 3h). The combination of rHBP and rMPO significantly increased vascular leakage in mice, which was more significant than that in the rHBP group or rMPO group (Figure 3i). Video evidence showed that the combination of rHBP and rMPO resulted in significant vascular leakage after 2 min (Video S5), which occurred much earlier than that in the rHBP group (approximately 7 min) (Video S4) or rMPO group (approximately 11 min) (Video S6).

### The MPO catalytic product HOCl but not MPO triggers CD44 extracellular domain shedding from vascular endothelial cells to damage the glycocalyx

**Figure 4.**
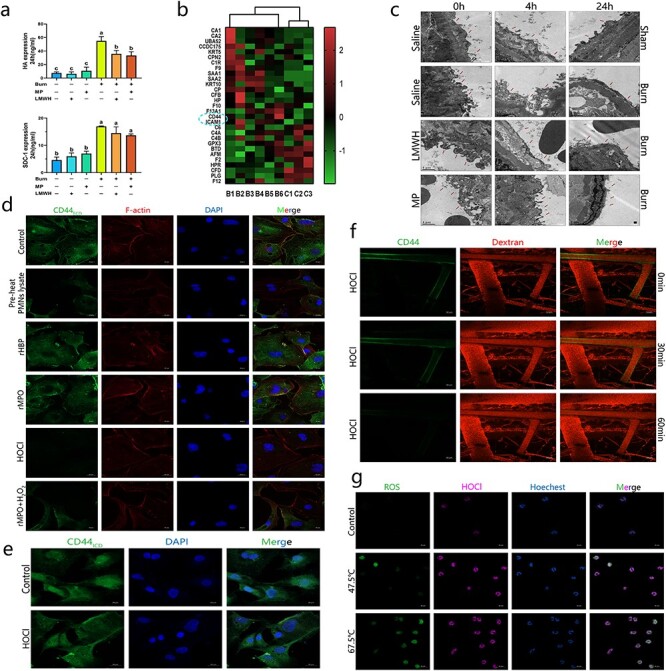
MPO catalytic product, HOCl, but not MPO, trigger CD44 extracellular domains shedding from vascular endothelial cells to damage glycocalyx. **(a)** A 30% TBSA rat burn model was used here. The blood samples of each rat were used for all the experiments shown in (a) and Figure S3a–c. LMWH, 200 IU/kg; MP, 30 mg/kg. Data are mean ± SD ANOVA with Tukey’s test. a, b, c: different letters represent significant differences between the two groups, *p* < 0.05. **(b)** The plasma samples of 6 severe burn patients and 3 healthy volunteers were analyzed by proteomics. Plasma collected from burn patients within 24 h after the burn (B, severe burn patient; C, healthy control). **(c)** The data showed the results of transmission electron microscopy of rat pulmonary veins, representing 4 independent experiments (Scale bar: 1 μm). **(d–g)** Pre-heat PMNs lysate, 67.5°C, 60 seconds, lysis by ultrasound. rHBP, 20 nM; rMPO, 20 nM; HOCl, 1 uM; H_2_O_2_, 1 uM. (d) and (e) used HMEC-1 cell line. (g) Thermal stimulation duration 60 seconds, human peripheral blood neutrophils. Micrographs are representative of >8 separate experiments (Scale bar: 20 μm). *CD44_ECD_* CD44 extracellular domain, *CD44_ICD_* CD44 intracellular domain, *HA* hyaluronic acid, *HOCl* hypochlorite, *ICAM-1* intercellular adhesion molecule-1, *LMWH* low molecular weight heparin, *MP* methylprednisolone, *rHBP* recombinant HBP, *rMPO* recombinant MPO, *ROS* reactive oxygen species, *SDC-1* syndecan-1, *TBSA* total body surface area

Glycocalyx decomposition products in the plasma of burn-injured rats were analyzed. HA, HS and SDC-1 in the burn-injured group were significantly higher than those in the sham group at 24 h (Figure S3a). Peripheral blood was collected from rats, and total plasma protein and albumin were measured in each group. The data showed that the neutrophils from severe burn-injured rats increased progressively within 24 h, while the plasma total protein and albumin levels decreased progressively (Figure S3c). Transmission electron microscopy analysis of rat pulmonary veins showed that low molecular weight heparin (LMWH) or methylprednisolone had a partial protective effect on the glycocalyx in severe burn-injured rats (Figure 4c). ELISA analysis showed that only HA was significantly downregulated by LMWH and methylprednisolone (Figure 4a, and Figure S3b). We performed proteomic analysis of the plasma of 6 severe burn patients and 3 healthy volunteers—and the data showed that the expression of CD44 and ICAM-1 was upregulated in the early stage of severe burn compared with that in the control group (Figure 4b). Neutrophils were stimulated by transient thermal stimulation, and the lysate of pre-heat-stimulated neutrophils significantly reduced the expression of CD44 in HMEC-1 cells, while neutrophil-derived lysate without thermal stimulation did not exert this effect. The culture supernatant of thermally stimulated neutrophils significantly downregulated the expression of CD44 in HMEC-1 cells, while the culture supernatant of unstimulated neutrophils did not. Cell lysate-mediated inhibition was more obvious than that of the cell culture supernatant (Figure S3d). Co-culture of HMEC-1 cells with thermally stimulated neutrophils significantly downregulated the expression of CD44 in HMEC-1 cells in a concentration-dependent manner, while unstimulated neutrophils did not have this effect (Figure S3e). An HBP inhibitor (aprotinin), MPO inhibitor (AZD5904), NE inhibitor (sivelestat), and MMP inhibitor (ilomastat) were added to the thermally stimulated neutrophil-derived lysate, and then HMEC-1 cells were stimulated. Only AZD5904 partially rescued the cell lysate-induced downregulation of CD44. rHBP, rMPO, nNE and rMMP9 were used to stimulate HMEC-1 cells, and only rMPO significantly downregulated the expression of CD44, but this effect was significantly weaker than that of cell lysates (Figure S3f). These data suggest that it is the catalytic substrate or product of MPO, not MPO itself, that downregulates the expression of CD44 in HMEC-1 cells (Figure S3g).

Immunofluorescence analysis of the CD44 extracellular domain of HMEC-1 cells showed that thermally stimulated neutrophil-derived lysate or HOCl could significantly reduce the fluorescence intensity of the CD44 extracellular domain. A similar effect was observed when rMPO and H_2_O_2_ were added together, but rHBP or rMPO alone had no similar effect (Figure 4d). After stimulation with 10 μM HOCl, the intracellular domain of CD44 in HMEC-1 cells was stained, and the fluorescence intensity did not decrease significantly (Figure 4e). After staining for CD44 on the vascular endothelial cells of living mice, HOCL was injected into the mouse tail vein. Compared with that in the control group (Figure S3h), a significant decrease in fluorescence intensity was observed within 60 min (Figure 4f). Fluorescent probes were used, and the production of intracellular ROS and HOCL increased significantly after the thermal stimulation of neutrophils (Figure 4g). Because the emission wavelength of the HOCl probe was too similar to that of Hochster fluorescent dyes, the nuclei showed some nonspecific staining. Interestingly, both rHBP and rMPO significantly inhibited the transcription of CD44 in HMEC-1 cells within 48 h, but the inhibitory effect of rHBP decreased gradually over time, while the inhibitory effect of rMPO increased gradually (Figure S3i).

### Damage to the glycocalyx results in firm adhesion of neutrophils and increases vascular leakage

**Figure 5.**
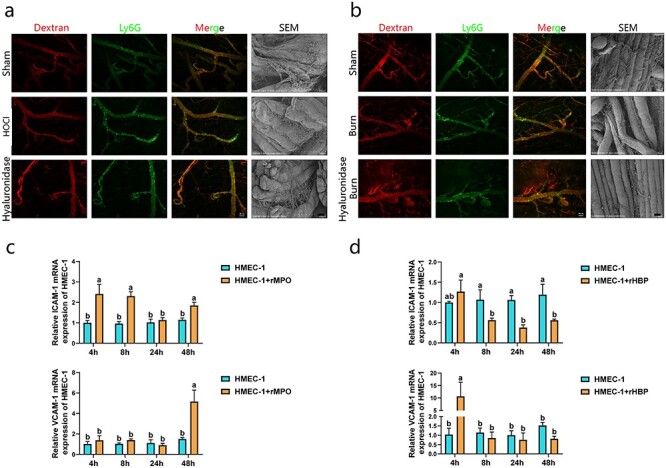
Damage to the glycocalyx result in firm adhesion of neutrophils and increase vascular leakage. (**a**, **b**) Hyaluronidase and burn group: hyaluronidase was injected into tail vein (diluted with normal saline), and mice burn model was established 1 h later. Hyaluronidase, 10 units/g; HOCl, 1 uM. Micrographs are representative of >8 separate experiments (Scale bar: 50 μm; Scale bar: 100 μm). (**c**, **d**) HMEC-1 cells were cultured to complete fusion. n = 4. rMPO, 20 nM; rHBP, 20 nM. Data are mean ± SD Differences were analyzed by repeated measurement ANOVA, compared with every other group. ^*^^*^^*^^*^p < 0.0001, ^*^^*^^*^p < 0.001, ^*^^*^p < 0.01, ^*^p < 0.05. a, b: different letters represent significant differences between the two groups, *p* < 0.05. *HMEC-1* human microvascular endothelial cells, *HOCl* hypochlorite, *ICAM-1* intercellular adhesion molecule-1, *rHBP* recombinant HBP, *rMPO* recombinant MPO, *SEM* scanning electron microscope, *VCAM-1* vascular cell adhesion molecule-1

The data showed that neutrophils in the sham group flowed freely in the blood vessels of the abdominal wall of the mice, and a large number of neutrophils adhered to the vessel wall after the administration of HOCl. A similar effect was observed after glycocalyx damage by hyaluronidase (Figure 5a, Videos S7, S8, and S9) [36, 37]. Scanning electron microscopy showed that the vascular barrier in the sham group was intact, and there was no obvious cell adhesion on the vascular wall. The vascular barrier in the HOCl or hyaluronidase group was thinner than that in the sham group, and a large number of cells adhered to the vascular wall (Figure 5a). *In vivo* vascular imaging and scanning electron microscopy data of mice showed a similar effect in the burn group as in the HOCl and hyaluronidase groups: the vascular barrier decreased significantly, and a large number of cells adhered to the vascular wall. After the glycocalyx was destroyed by hyaluronidase, vascular leakage and neutrophil adhesion were more significant in burn-injured mice (Figure 5b). PCR analysis showed that rMPO-stimulated HMEC-1 cells significantly upregulated ICAM-1 transcription within 8 h and VCAM-1 transcription at 48 h. After rHBP stimulation of HMEC-1 cells, the transcription of VCAM-1 was significantly upregulated within 4 h (Figure 5c, d).

### MPO inhibitors partially protect the glycocalyx in the early stage of severe burns. The combination of HBP and MPO inhibitors reduces vascular leakage and systemic edema

**Figure 6.**
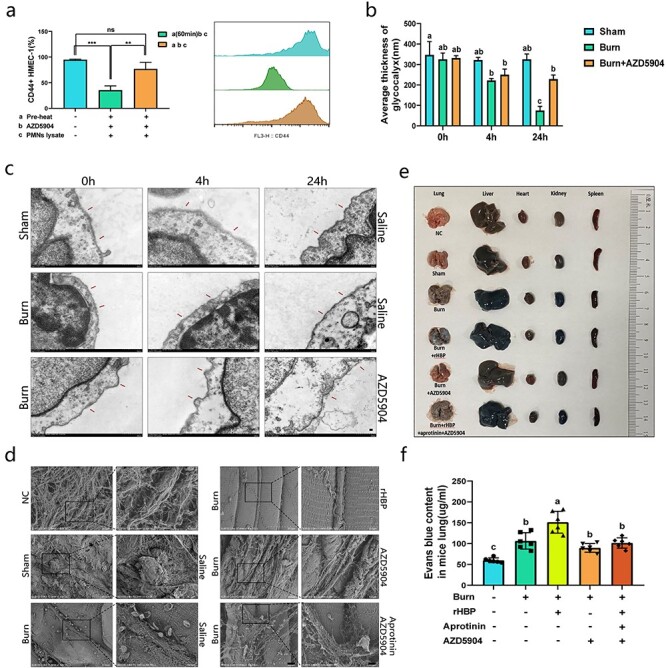
MPO inhibitor showed partial protection for glycocalyx in the early stage of severe burns. The combination of HBP and MPO inhibitors reduce vascular leakage and systemic edema. **(a)** a (60 min); b, c: After thermal stimulation, neutrophils were incubated at 37°C for 60 min and then added to AZD5904, and cell lysate was obtained 15 min later; a, b, c: After thermal stimulation, neutrophils were added to AZD5904, washed off the inhibitor 15 min later, and continued to culture at 37°C. Thermal stimulation: 67.5°C, 60 seconds. Both experiments to take the same time from the end of thermal stimulation to obtaining cell lysate. n = 4. **(b, c)** Transmission electron microscopy of femoral veins in mice. Micrographs are representative of >8 separate experiments. The thickness of glycocalyx was measured by Hitachi TEM system (Scale bar: 500 nm). a, b, c: different letters represent significant differences between the two groups, *p* < 0.05. **(d)** Scanning electron microscope of femoral veins in mice. Micrographs are representative of >8 separate experiments (Scale bar: 30 um, 10 um). **(e, f)** Evans blue solution (50 μl of a 30 mg/ml solution in 0.9% normal, unheparinized saline) was injected via tail veins of mice. rHBP, aprotinin and AZD5904 were injected into tail vein before the establishment of burn model. n = 6 in each group. rHBP, 1.5ug/g; aprotinin, 2.5ug/g; AZD5904, 0.5ug/g. Photo is representative of 6 separate experiments of mice *in vivo*. Data are mean ± SD ANOVA with Tukey’s test. ^*^^*^^*^p < 0.001, ^*^^*^p < 0.01; a, b: different letters represent significant differences between the two groups, *p* < 0.05. *NC* non-specific control, *rHBP* recombinant HBP

Two different procedures were performed. (1) After thermal stimulation, neutrophils were incubated at 37°C for 60 min, AZD5904 was added, and the cell lysate was obtained after 15 min. (2) After thermal stimulation, AZD5904 was added to neutrophils, the inhibitor was washed off 15 min later, and the cells were cultured at 37C for 1 h and then lysed. Flow cytometry showed that the former treatment had a significant protective effect on CD44 (Figure 6a). Transmission electron microscopy analysis of mouse femoral veins showed that AZD5904 had a partial protective effect on the glycocalyx after burn injury (Figure 6b, c). Scanning electron microscopy showed that there were many interwoven vascular barriers on the surface of femoral veins in the untreated and sham groups, and no obvious cells adhered to the vessels. In the burn-injured group and the burn-injured plus rHBP group, the vascular barrier was significantly reduced and loose, with a large number of cells adhering to the vascular wall. Compared with the AZD5904 group, the AZD5904 plus aprotinin group showed no additional protective effect on the vascular barrier (Figure 6c). After the same intervention as that shown in Figure 6d, the mice were stained with Evans blue. The data showed that early administration of AZD5904 could reduce vascular leakage and systemic edema, but this effect was not significant. Compared with the burn-injured group, the AZD5904 plus aprotinin group did not exhibit significantly inhibition of vascular leakage and systemic edema. Compared with that in the burn-injured plus rHBP group, the combined use of these inhibitors significantly inhibited vascular leakage and tissue edema (Figure 6e, f).

## Discussion

In the early stage of severe burns, patients have severe shock. How to provide the best fluid resuscitation in the first 24 to 48 h after injury is the most difficult and controversial component of the treatment of burn patients [38]. After severe burn, a large amount of fluid transfers from blood vessels to tissue space, resulting in decreased blood volume and cardiac output, forming a vicious circle and aggravating shock symptoms. Conversely, rapid and massive fluid resuscitation easily becomes excessive, resulting in a series of complications, including pulmonary edema, gastrointestinal edema and abdominal compartment syndrome [39, 40]. In the past few decades, many possible factors have been proposed, including lipid mediators [41], peroxides [42], substance P [43, 44], bradykinin [45], histamine [33] and HBP [31, 46], each of which may induce vascular endothelial cell leakage. However, there is still a lack of in-depth studies on the mechanism of vascular leakage and systemic edema in the early stage of severe burns. Here, we provide evidence that systemic edema caused by vascular leakage in the early stage of severe burns is triggered by neutrophil-derived HBP and MPO.

We observed that in addition to obvious systemic edema, the expression of multiple neutrophil-derived proteins and glycocalyx decomposition products increased significantly in the early stage of severe burns. Through correlation analysis of these proteins and molecules, it is hypothesized that early edema and glycocalyx damage in severe burns may be associated with the abnormal secretion of HBP and MPO by neutrophils. Previous reports have shown that HBP and MPO are also upregulated in trauma and inflammation [47], but there is no systemic edema in these conditions, as there is in the early stages of severe burns. Our data show that there is a higher correlation between HBP and MPO in the early stage of severe burn than in severe trauma. The synchronous increase in HBP and MPO may be the cause of systemic edema in severe burns. Previous studies have confirmed that HBP can increase the permeability of vascular endothelial cells *in vivo* and *in vitro* [31, 48], which is consistent with our observation. Interestingly, although mice, rats and hamsters do not express HBP homologous genes, rHBP can significantly induce microvascular leakage in living hamsters. A reasonable explanation is that HBP is an evolutionarily conserved protein. Although mice do not express HBP homologous genes, they express homologous receptors of these genes. Thus, the addition of HBP to the blood of burn-injured mice is similar to the endovascular state of severely burned patients. In human microvascular endothelial cells and mice, we also demonstrated that HBP enhanced permeability and triggered vascular leakage, which was consistent with previous studies on other models. By constructing a transient thermal stimulation model, we showed that a single transient thermal stimulation could significantly trigger neutrophils to release HBP and MPO. Evans blue staining and wet/dry ratio analysis showed that vascular leakage triggered by HBP could cause significant systemic edema. A large number of blood vessel images and videos show that the combined use of HBP and MPO triggers fast and significant blood vessel leakage in living mice. To study the role of MPO in vascular leakage, we considered whether MPO destroyed the glycocalyx based on the analysis of MPO and glycocalyx destruction products. We found that both LMWH and methylprednisolone could reduce the expression of HA in the plasma of burn-injured rats, and the transmission electron microscopy results also showed that these factors had partial protective effects on the glycocalyx. Previous studies have confirmed that heparin can efficiently bind to HBP without affecting the binding of HBP to lipopolysaccharide (LPS) [49, 50]. Furthermore, as one of the most negatively charged molecules, LMWH can bind to many positively charged proteins and affect their functions. There have also been reports showing that methylprednisolone can inhibit neutrophil degranulation by downregulating neutrophil CD18 expression and regulating the PI3K pathway. Based on these data, we hypothesize that some positively charged proteins secreted by neutrophils may be associated with glycocalyx destruction.

Proteomic analysis of the plasma of 6 patients with severe burns within 24 h of injury revealed that the expression of CD44 and ICAM-1 was upregulated, and CD44 is the extracellular receptor of hyaluronic acid. We hypothesize that neutrophil-derived positively charged proteins mediate glycocalyx destruction by regulating CD44 expression. HMEC-1 cells were stimulated with different proteins and molecules, and we found that the catalytic substrate of MPO, HOCl, but not MPO itself could trigger CD44 extracellular domain shedding to damage the glycocalyx. Another interesting finding is that both rHBP and rMPO can significantly downregulate the transcription of CD44 in HMEC-1 cells within 48 h, but the effect of HBP gradually decreases over time, while the effect of MPO gradually increases. Immunofluorescence analysis showed that the production of HOCl in thermally stimulated neutrophils was significantly increased. HOCl could damage the glycocalyx, cause neutrophils to adhere to the vascular wall, and amplify the regulatory effect of neutrophil-derived HBP and MPO on vascular endothelial cells. Moreover, PCR analysis showed that MPO and HBP rapidly and significantly upregulated ICAM-1 and VCAM-1 mRNA levels, respectively, which partly explains the abnormal adhesion of neutrophils after glycocalyx injury [51, 52]. The combination of MPO and HBP inhibitors showed a significant protective effect on the glycocalyx in burn mice but only reduced vascular leakage and systemic edema to a certain extent. Compared with those in the burn-injured rHBP group, the combination of HBP and MPO inhibitors significantly reduced vascular leakage and systemic edema. This effect can be explained by the incomplete inhibition of the inhibitors, deletion of mouse HBP homologous genes and some unknown methods of regulating vascular permeability [31]. In the future, we will continue to conduct in-depth research by constructing HBP-humanized mice and developing HBP-specific inhibitors.

## Conclusions

In summary, this work comprehensively characterizes a new mechanism by which neutrophil-derived HBP and MPO regulate vascular endothelial cells and the glycocalyx, respectively, through different pathways. Neutrophil-derived HBP can trigger vascular leakage, while the MPO-catalyzed product HOCl can trigger CD44 shedding from vascular endothelial cells to damage the glycocalyx. The destruction of the glycocalyx induces abnormal neutrophil adhesion and increased vascular leakage. The synergistic effect of HBP and MPO induces vascular leakage and leads to systemic edema. Our findings indicate that neutrophil-derived HBP and MPO play important roles in vascular leakage and systemic edema in the early stage of severe burns. Targeting these two molecules may lead to new strategies for the treatment of severe burns.

## Supplementary Material

revised_supplemental_materials_tkab030

Video_S1_tkab030

Video_S2_tkab030

Video_S3_tkab030

Video_S4_tkab030

Video_S5_tkab030

Video_S6_tkab030

Video_S7_tkab030

Video_S8_tkab030

Video_S9_tkab030

## Data Availability

Data is available from the authors upon reasonable request.

## Abbreviations

BSA-HRP: HRP labeled bovine serum albumin; ECM: endothelial cell culture medium; h: hour; HA: hyaluronic acid; HBP: heparin binding protein; HMEC-1: human microvascular endothelial cells; HOCl: hypochlorite; HS: heparan sulfate; ICAM-1: intercellular adhesion molecule-1; LMWH: low molecular weight heparin; LPS: lipopolysaccharide; MAP: mean venous pressure; MMP9: matrix metallopeptidase-9; MPO: myeloperoxidase; NE: neutrophil elastase; PBS: phosphate buffered saline; PMN: polymorphonuclear leukocyte; rHBP: recombinant HBP; rMPO: recombinant MPO; ROS: reactive oxygen species; SDC-1: syndecan-1; TBSA: total body surface area; VCAM-1: vascular cell adhesion molecule-1; qRT-PCR: quantitative real time polymerase chain reaction; FBS: fetal bovine serum; ECGS: endothelial cell growth supplement; P/S: penicillin-streptomycin; OD: optical density; TMB: tetraMethyl benzidine.
