# Supplementary material for: Neutrophil-derived heparin binding protein triggers vascular leakage and synergizes with myeloperoxidase at the early stage of severe burns (With video)
Source: Burns Trauma. 2021 Sep 17;9:tkab030. doi: 10.1093/burnst/tkab030 (PMC8499692; doi:10.1093/burnst/tkab030)
Supplement: revised_supplemental_materials_tkab030 [file revised_supplemental_materials_tkab030.docx]

**Supporting Information for**

**Neutrophil-derived HBP triggers vascular leakage in MPO synergy at the early stage of severe burns**

Lu Liu, Yiming Shao, Yunxi Yang, Yixuan Zhang, Jiamin Huang, Linbin Li, Ran Sun, Yuying Zhou, Yicheng Su, Bingwei Sun**^*^**

**^*^** **Corresponding author**

Email: [sunbinwe@hotmail.com](mailto:sunbinwe@hotmail.com)

**This file includes:**

Supplementary text

Figures S1 to S3

Video S1 to S9

**
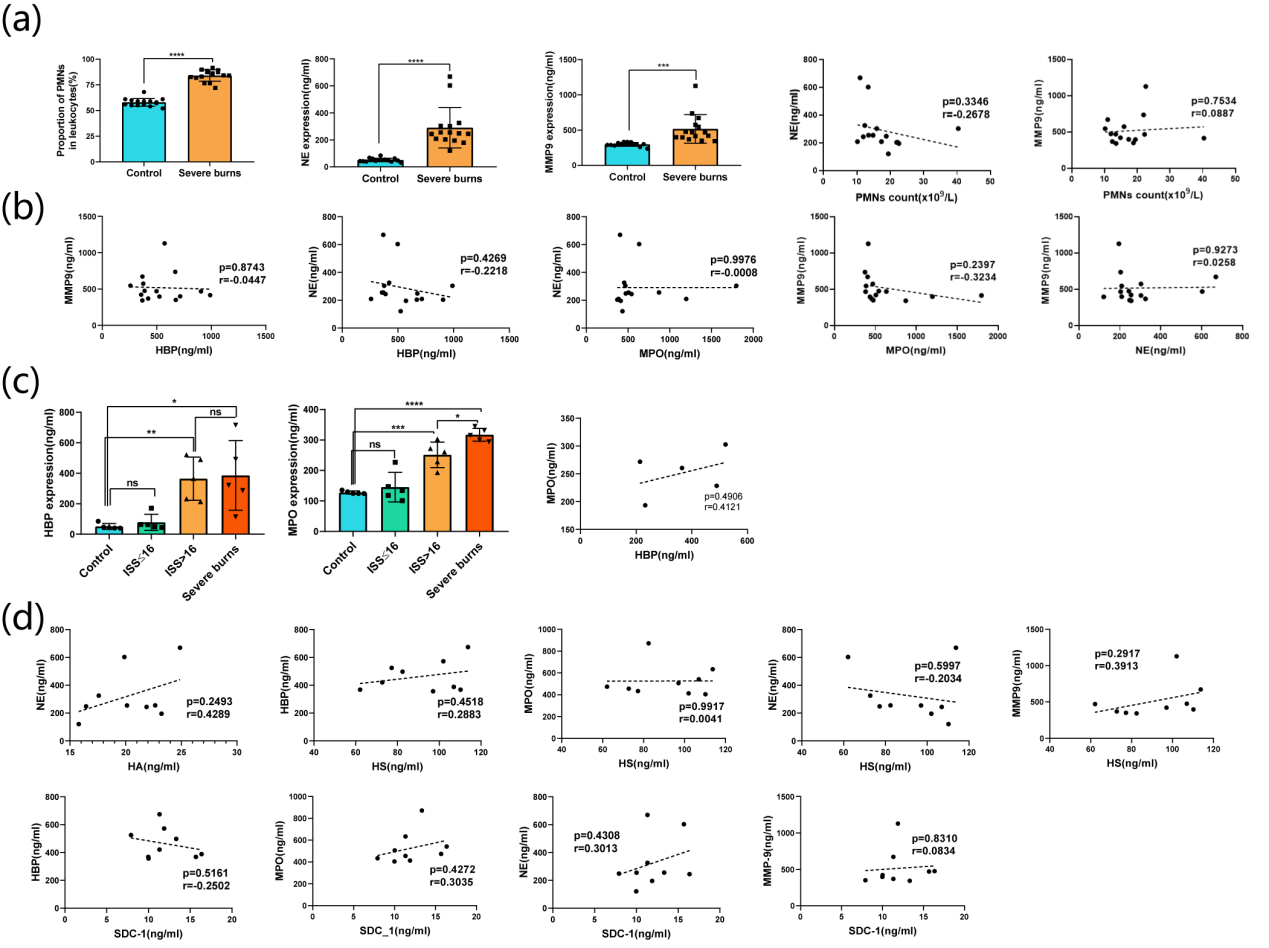
**

**Figure S1. (a)** The proportion of neutrophils, the expression of NE and MMP9 in plasma of 15 severe burn patients corresponding to Figure 1 were supplemented to show. The correlation analysis between NE and MMP9, and the count of neutrophils did not show a significant correlation. **(b)** As a supplement to figure 1B, the correlation analysis between HBP, MPO, NE and MMP9 was performed in pairs, no correlation was shown except between HBP and MPO. **(c)** 10 non-burn trauma patients were divided into two groups based on the injury severity score (ISS)-. All samples including the healthy control group and the severe burn group were collected within two weeks. All plasma is tested in one day. **(d)** As a supplement to figure 1F. There was no significant difference in all the correlation analysis as shown in the figure. All the samples in figure S1 were from healthy volunteers, burn patients and trauma patients. Independent sample t test was used to prove the difference between two groups. ****, *p*<0.0001, ***，*p*<0.001, **, *p*<0.01, *, *p*<0.05, Compared with control group. MMP9: matrix metalloprotein-9; NE: neutrophil elastase; PMNs: polymorphonuclear leukocytes; HBP: heparin binding protein; MPO:myeloperoxidase; ISS: Injury severity score;HA: hyaluronic acid; HS: heparan sulfate; SDC-1: syndecan-1.


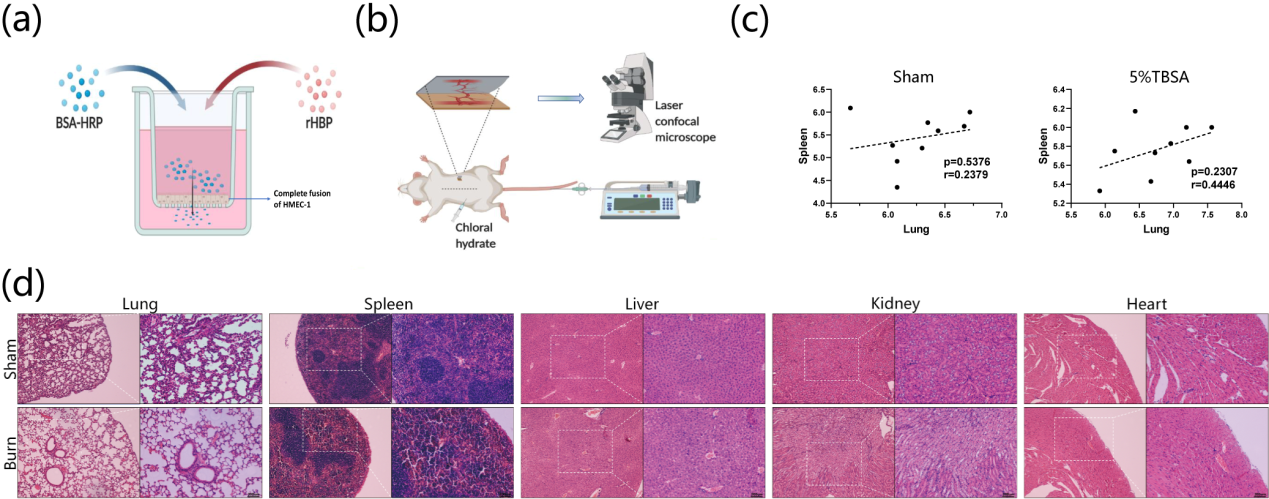


**Figure S2. (a)**The seeding density of HMEC-1 cells in transwell-24 system was 5 × 10^5^/ ml and 100uL per well. The cells were completely fused by ECM culture. **(b)** Mice were anesthetized with 8% chloral hydrate at a dose of 10ul / g.Zeiss LSM 900 confocal microscope was used to observe the skin after aseptic operation. **(c)** Correlation analysis of wet dry ratio of spleen and lung in burn group and 5% TBSA group. n=9. **(d)** HE staining of lung, spleen, liver, kidney and heart in 20% TBSA group.Micrographs are representative of >9 separate experiments.BSA-HRP: HRP labeled bovine serum albumin; rHBP:recombinant HBP; TBSA: total body surface area.


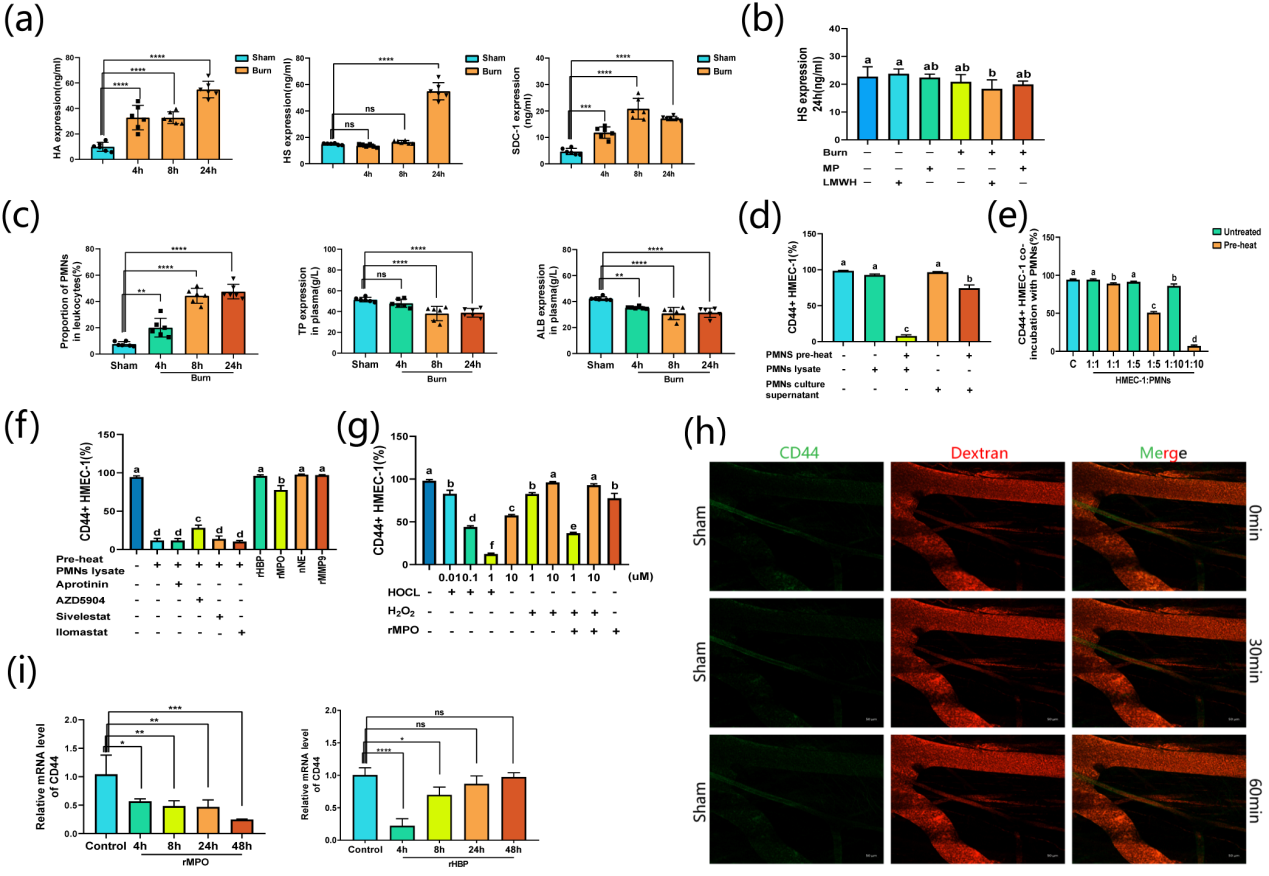


**Figure S3. (a-c)** As a supplement to figure 4. A, n=6 in each group. **(d)** Flow cytometry was used. PMN pre-heat: Human peripheral blood PMNs were incubated at 37 ℃ for 60 minutes after a 60 seconds heat stimulation at 67.5 ℃. **(e)** Pre-heat: Human peripheral blood PMNs were stimulated at 67.5 degrees for 60 seconds, and then incubated with HMEC-1 cells. **(f) (g)** Flow cytometry was used. Pre-heat PMNs lysate: Human peripheral blood PMNs were heated at 67.5 ℃ for 60 seconds, incubated at 37 ℃ for 60 minutes, and then lysed with ultrasound. Aprotinin, 20ug/ml; AZD5904, 500nM; Sivelestat, 200nM; Ilomastat, 100nM; rHBP, 20nM; rMPO, 20nM; nNE, 1ug/mL; rMMP9, 1ug/ml. **(h)** Control group of figure 4F. Micrographs are representative of >8 separate experiments. Scale bar, 20 µm. **(i)** HMEC-1 cells were cultured to complete fusion. Data are representative of four (d-g, i) independent experiments. Data are mean ± s.d.; n = 4. ANOVA with Tukey’s test. ****, *p*<0.0001, ***，*p*<0.001, **, *p*<0.01, *, *p*<0.05; a, b, different letters represent significant differences between the two groups, *p*<0.05. HA: hyaluronic acid; HS: heparan sulfate; SDC-1: syndecan-1; MP: methylprednisolone; LMWH: low molecular weight heparin; TP: total protein; ALB: albumin; PMNs: polymorphonuclear leukocytes; HMEC-1: human microvascular endothelial cells; rMPO: recombinant MPO; HOCL: hypochlorite.


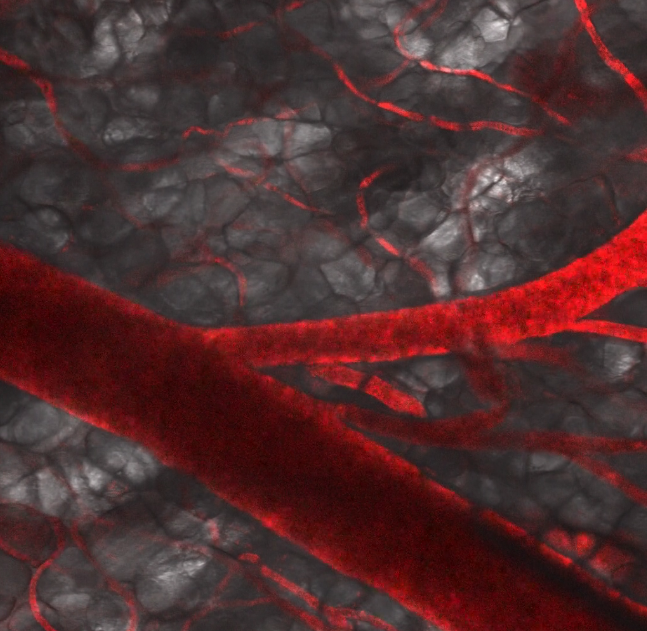


**Supplemental video S1. Dynamic observation of blood flow in mice.** Texas Red dextran was injected through the tail vein of mice as a plasma tracer at a dose of 25ug/g. Molecular weight of Texas Red dextran, 70,000MW. The video represents a 30-minute shot. Zeiss LSM 900 confocal microscope was used to observe the blood flow. Scale bar, 50um.


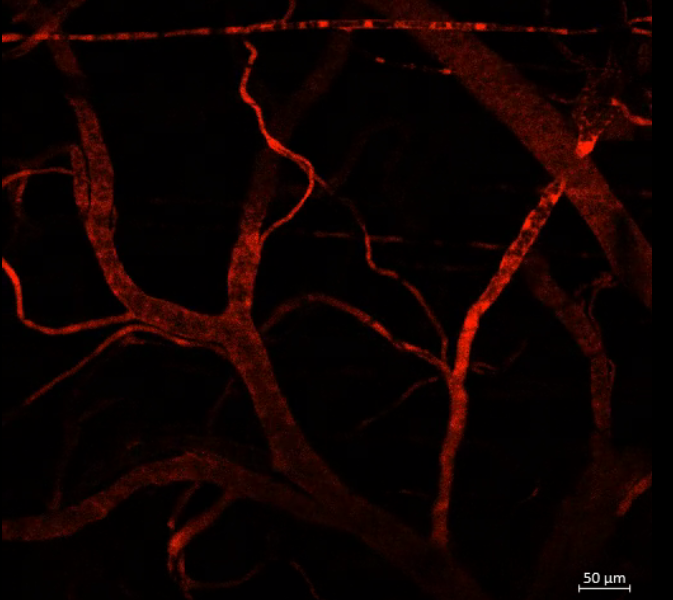


**Supplemental video S2. Vascular leakage was not observed in the sham group.** The video represents a 30- minute shot. Zeiss LSM 900 confocal microscope was used for observation. Scale bar, 50um.


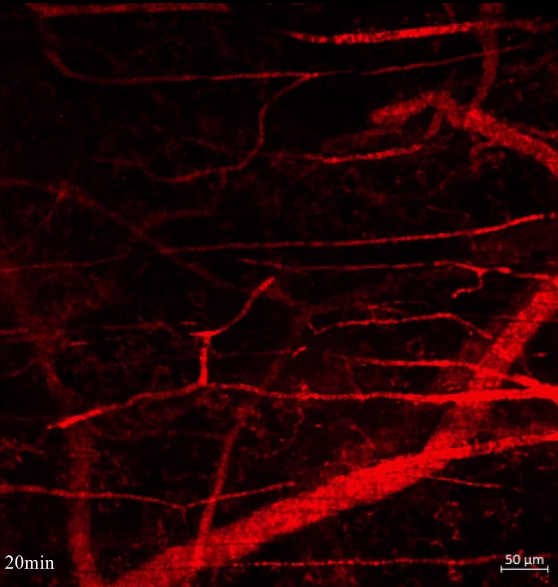


**Supplemental video S3. Significant vascular leakage was observed in burn-injured mice.** The video represents a 40-minute shot. This video is a 40 minutes shot, showing the vascular leakage of mice from 5 h 40 to 6 h 20 after burn. Zeiss LSM 900 confocal microscope was used for observation. Scale bar, 50um.


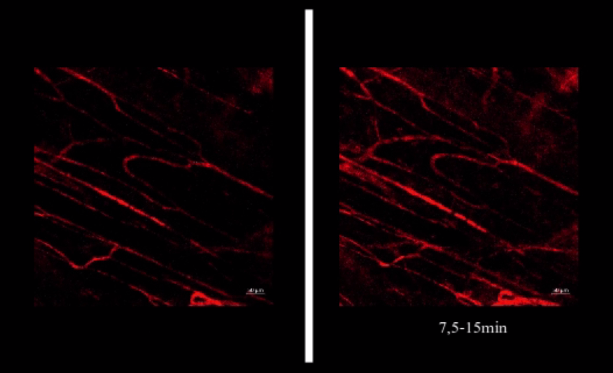


**Supplemental video S4. Recombinant HBP significantly triggered vascular leakage of mice.** The video represents a 15-minute shot showing significant vascular leakage after injection of rHBP into the tail vein of mice. Zeiss LSM 900 confocal microscope was used for observation. Scale bar, 50um.


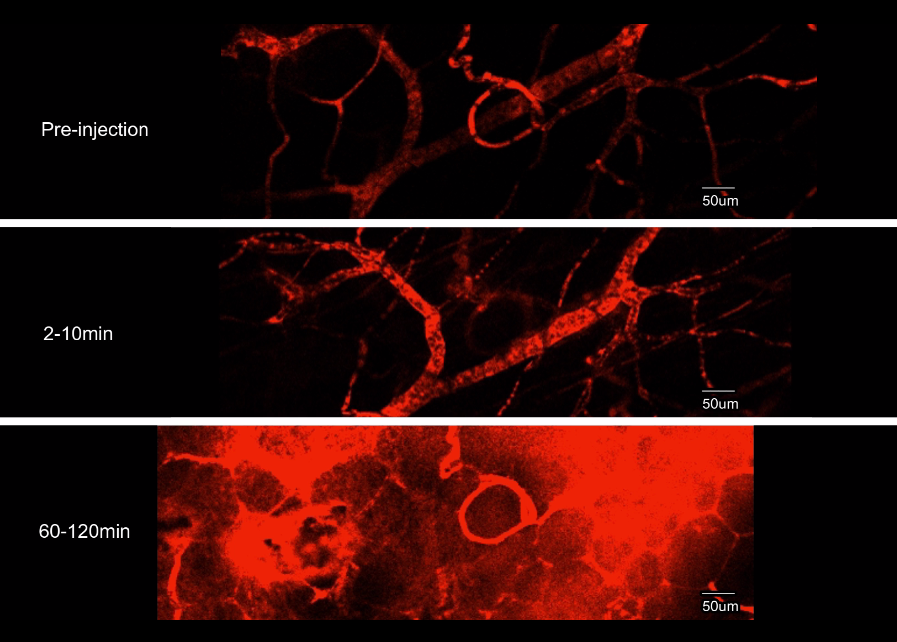


**Supplemental video S5. The combination of rHBP and rMPO significantly triggered vascular leakage in mice.** This video represents a 120-minute shot showing obvious vascular leakage after simultaneous injection of rHBP and rMPO into the tail vein of mice. Zeiss LSM 900 confocal microscope was used for observation. Scale bar, 50um.


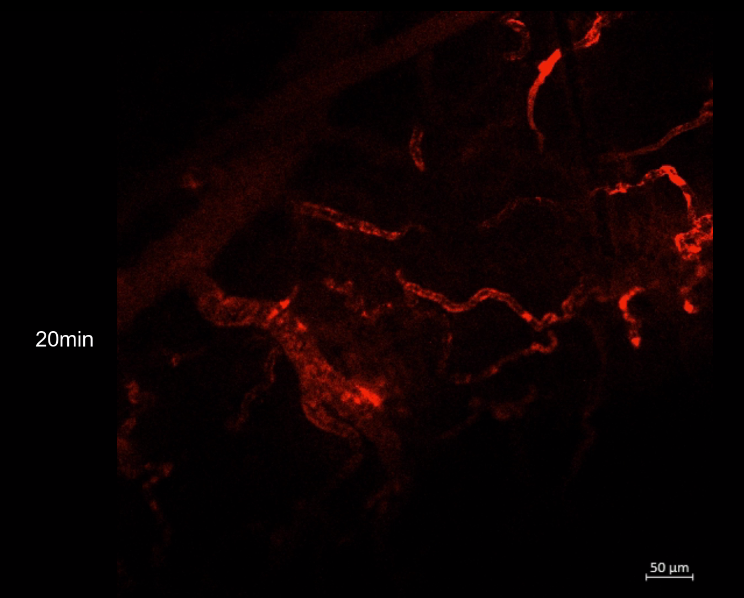


**Supplemental video S6. Recombinant MPO significantly triggered vascular leakage of mice.** The video represents a 20-minute shot showing significant vascular leakage after injection of rMPO into the tail vein of mice. Zeiss LSM 900 confocal microscope was used for observation. Scale bar, 50um.


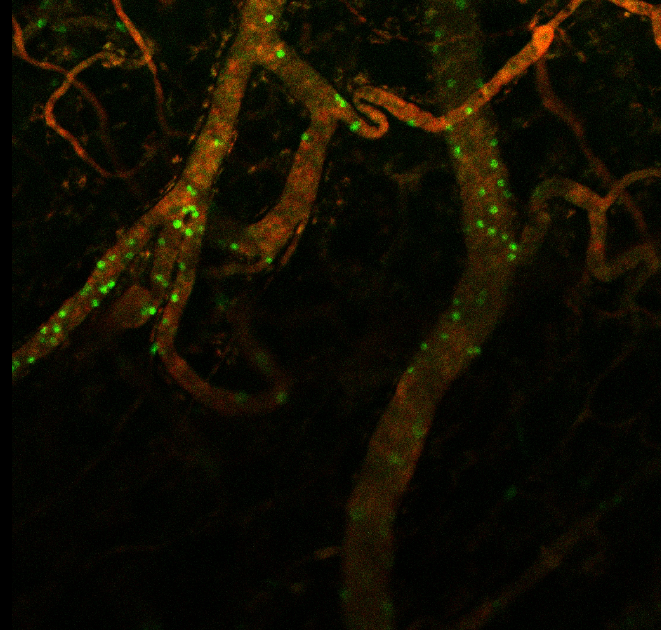


**Supplemental video S7. Movement of neutrophils in blood vessels in sham group.** The video represents a 40-second shot showing that neutrophils in the sham group move with the blood flow and less adhere to the vessel wall. Neutrophils of mice was labeled with Biotin anti-mouse Ly-6G and iFluor™ 647-streptavidin conjugate. The blood color shown is a mixture of Texas Red and iFluor™ 647. Zeiss LSM 900 confocal microscope was used for observation. Scale bar, 50um.


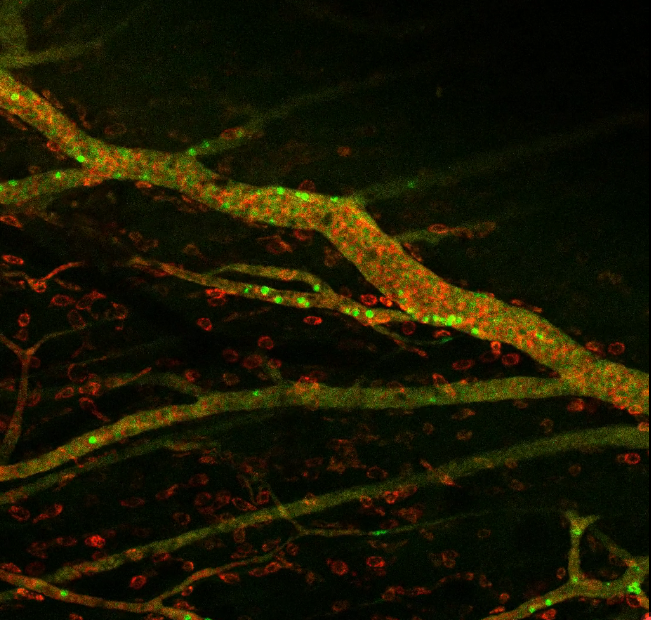


**Supplemental video S8. Neutrophils in the HOCl treated group adhered to the vessel wall**. The video represents an 80-second shot showing a large number of neutrophils adhering to the vessel wall after HOCl treatment. Neutrophils of mice was labeled with Biotin anti-mouse Ly-6G and iFluor™ 647-streptavidin conjugate. Zeiss LSM 900 confocal microscope was used for observation. Scale bar, 50um.


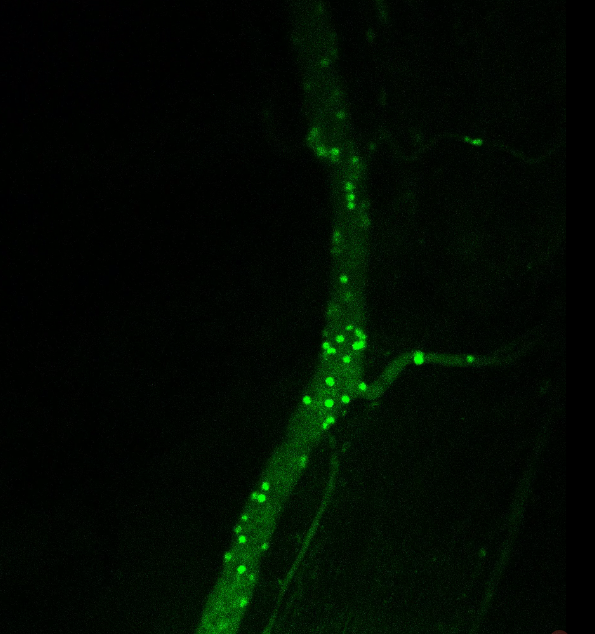


**Supplemental video S9. Neutrophils in the hyaluronidase treated group adhered to the vessel wall**. The video represents a 100-second shot showing a large number of neutrophils adhering to the vessel wall after hyaluronidase treatment. Neutrophils of mice was labeled with Biotin anti-mouse Ly-6G and iFluor™ 647-streptavidin conjugate. Zeiss LSM 900 confocal microscope was used for observation. Scale bar, 50um.
